# Identifying emergency presentations of chronic liver disease using routinely collected administrative hospital data

**DOI:** 10.1016/j.jhepr.2024.101322

**Published:** 2024-12-31

**Authors:** Jessica King, Vikram Bains, James Doidge, Jan Van Der Meulen, Kate Walker, William Bernal

**Affiliations:** 1 Department of Health Services Research & Policy, London School of Hygiene & Tropical Medicine, London, UK; 2 Clinical Effectiveness Unit, Royal College of Surgeons of England, London, UK; 3 Liver Intensive Therapy Unit, Kings College Hospital, London, UK; 4 Institute of Liver Studies, King’s College London, University of London, London, UK; 5 Intensive Care National Audit and Research Centre, London, UK

**Keywords:** Cirrhosis, Electronic health records, Emergency care, Phenotype

## Abstract

**Background & Aims:**

Patients with chronic liver disease (CLD) are often first diagnosed during an emergency hospital admission, when their disease is advanced and survival is very poor. Evaluating their care and outcomes is a clinical research priority, but methods are needed to identify them in routine data.

**Methods:**

We analysed national administrative hospital data in the English National Health Service. We used existing literature, expert clinical opinion, and data-driven approaches to develop three algorithms to identify first-time emergency admissions in 2017–2018. We validated these in 2018–2019 data by assessing the distributions of predictive factors, treatments, and outcomes associated with CLD in the patients captured by each algorithm.

**Results:**

Our most specific algorithm identified 10,719 patients with CLD who first presented through emergency hospital admission from April 2018 to March 2019. Alternative, less specific or more sensitive algorithms identified 12,867 or 20,828 patient, respectively. Additional patients identified by more sensitive algorithms had more comorbidities, were less likely to die from CLD, and were less likely to be treated by a gastroenterologist or hepatologist.

**Conclusions:**

Three algorithms are provided that successfully identified patients in administrative hospital data with a first emergency admission for CLD. The choice of algorithm should reflect the aims of the research.

**Impact and implications:**

The more and most sensitive algorithms are recommended in studies when it is important to minimise the number of patients with CLD erroneously missed from the cohort, such as studies measuring disease burden. The most specific algorithms might miss patients whose primary reason for admission is recorded as a sign, symptom, or complication of CLD, but is recommended when the interest is strictly in patients whose primary reason for emergency admission is CLD.

## Highlights

• People with CLD are often first diagnosed during an emergency hospital admission.
• We developed and validated three algorithms to identify these patients in routine data.
• The three algorithms identified 10,719, 12,867, and 20,828 patients per year, respectively.
• The most specific algorithm is recommended when the focus is strictly patients admitted for CLD.
• The more and most sensitive algorithms are recommended for studies of disease burden.

## Graphical abstract

**Figure:**
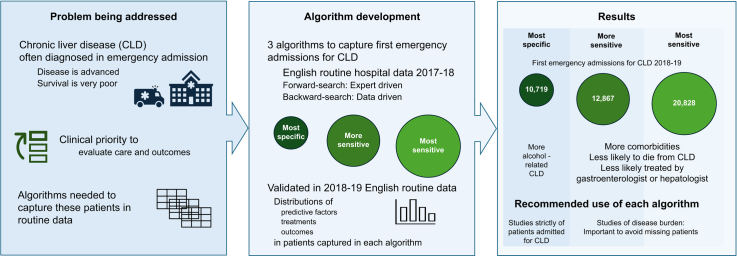

## Introduction

Chronic liver disease (CLD) is a major health concern in England and is increasing in prevalence.^1^ Deaths from CLD have risen almost fivefold since 1970, and most people with CLD die before they are 65 years old.^2^ In 2020, CLD was the second leading cause of mortality in 35–49-year-olds, accounting for 9.8% of deaths.^3^ The end-stage of CLD is cirrhosis (irreversible damage to the liver), a condition that typically develops over many years.^4^ However, people with CLD often first come to medical attention during an emergency admission to hospital, when their disease is already advanced and cirrhosis is present, often with decompensation and/or portal hypertension (advanced CLD; AdvCLD).^5^ Outcomes for these patients are very poor: a quarter will die within 60 days of their first emergency admission and nearly 70% of those who survive their admission will die within 5 years.^6^

Identifying emergency presentations of CLD in routinely collected administrative hospital data is a crucial first step in research measuring the burden of disease, healthcare resource use, or exploring questions about the clinical effectiveness and cost effectiveness of different treatment approaches. For example, up to one-third of people with CLD might require a transfer to a high-dependency unit (HDU) or intensive care unit (ICU) for critical care support, but there is evidence that not all patients who might benefit from critical care receive it.^7^^,^^8^ Similarly, access to specialist gastroenterology care is associated with longer survival, yet in 2018–2019, less than two-thirds of patients with liver disease admitted as an emergency in England were seen by a specialist in gastroenterology or hepatology.^9^ More evidence for the effectiveness of specialist gastroenterological involvement and critical care for patients with CLD is important to maximise its value in improving survival.^10^

Administrative hospital data, routinely collected through the normal operations of hospital organisations, are widely used for clinical, epidemiological, and health services research.^11^ Examples include the Medicaid^12^ and Veterans Affairs^13^ databases in the USA and the Saskatchewan Health Services Database in Canada.^14^ Hospital Episode Statistics (HES), used in this study, contain records of all patients admitted to all English National Health Service (NHS) hospitals, and uses a standard international system for coding clinical diagnoses.^15^

Identifying patients admitted to hospital for CLD in routinely collected administrative hospital data is not straightforward. First, CLD encompasses a range of diagnoses and can have several different aetiologies; thus, no single diagnosis can be used to identify it. Second, patients admitted for CLD might present with a variety of complications of CLD, including ascites, hepatic encephalopathy, and gastrointestinal bleeding. Even when CLD is the underlying condition leading to the admission, signs, symptoms, or complications of CLD might be recorded as the primary diagnosis, rather than CLD itself. Finally, patients admitted for CLD are often seriously ill, with multiple comorbidities,^16^ and it might be unclear whether CLD is the primary reason for admission, an incidental diagnosis, or a comorbidity.

Using routinely collected administrative hospital data, we developed and validated coding algorithms to identify patients with a first emergency hospital admission for CLD, and to provide recommendations for the use of the coding algorithms in CLD research.

## Methods

### Data

The study used HES data linked to Office for National Statistics (ONS) mortality data. Hospital admissions from April 2017 to March 2018 were used to develop the algorithms to identify first-time emergency hospital admissions for CLD. Hospital admissions from April 2018 to March 2019 were used to validate the algorithms. HES data from April 2013 onward were used to identify previous admissions for CLD. The study was reviewed and approved by the institutional ethics committee of London School of Hygiene and Tropical Medicine (LSHTM; Ref 28468). The legal basis for processing HES data is under Article 9.2 (h) of GDPR, for the purposes of preventive or occupational medicine, for the assessment of the working capacity of the employee, medical diagnosis, the provision of health or social care or treatment or the management of health or social care systems and services.

### Hospital episode statistics

HES is a national administrative dataset of all care provided in NHS hospitals.^15^ This study used admitted patient care (APC) data, which includes all records of day-case and overnight admissions. Each episode is a continuous period of care under one senior clinical specialist (‘consultant’) at one hospital. A patient can have multiple episodes within a single admission if their care is transferred between consultants. In HES, pseudo-identifiers enable episodes for the same patient to be linked longitudinally.

HES includes patient demographics, such as age and ethnicity, dates and times of admission and discharge, and information relating to diagnosis and management. For each episode, a primary diagnosis is recorded using the International Classification of Diseases, 10th revision (ICD-10).^17^ The primary diagnosis is defined as the main condition treated or investigated during the episode, or, if there is no definitive diagnosis, the main symptom, abnormal findings, or problem.^17^ Up to 19 secondary diagnoses can also be coded using ICD-10 to record other relevant diagnoses or comorbidities. Any procedures carried out are coded using the Office of Population Censuses and Surveys Classification of Surgical Operations and Procedures, 4th revision (OPCS-4).^18^ The RCS Charlson comorbidity score was used to determine the number of comorbid conditions for each patient in the first emergency admission or admissions during the preceding year,^19^ excluding liver disease and liver cancer. Information about patients’ socioeconomic status is available from the Index of Multiple Deprivation (IMD), an area-level measure of deprivation derived from information about income, education, employment, crime, and the living environment for areas of residence that typically include about 600 households or 1,500 individuals.^20^

ONS mortality data provided dates and causes of death from April 2017 to 20 September 2020.

### Algorithm development

Our approach to algorithm development had four steps: (1) forward searching: development of an initial set of diagnostic codes based on previous studies and expert clinical input; (2) backward searching: expansion of the initial set of diagnostic codes, using a data-driven approach to identify additional common diagnostic codes in patients likely to have had an emergency admission for CLD; (3) code classification: classification of each code into: (A) indications of cirrhotic CLD; (B) indications of non-cirrhotic CLD; (C) signs, symptoms, complications, or causes of CLD; or (D) codes that indicate that CLD is not the primary reason for admission; and (4) algorithm specification: definition of the algorithms according to combinations of categories of codes in the primary and secondary diagnosis fields.

#### Forward searching

A list was compiled of ICD-10 codes that are likely to be used to code CLD and its complications, based on previous studies that have used routinely collected administrative hospital data to identify CLD and cirrhosis,[21], [22], [23], [24], [25], [26], [27], [28], [29] deaths caused by cirrhosis,^30^ or CLD as a comorbidity,^31^ or based on expert clinical input (VB and WB).

#### Backward searching

Backward searching is a data-driven approach using sets of patients who are highly likely to have presented in an emergency with CLD, identified through means other than diagnosis codes. The aim was to pick up unpredictable idiosyncrasies of coding practices not included in the forward searching step. Patients were identified as likely to have been admitted to hospital for CLD if they had a procedure associated with AdvCLD, or if they died from CLD.

Three groups of procedures were examined: paracentesis, treatment of variceal bleeding, and transjugular intrahepatic portosystemic shunt (TIPS). Patients were included in the backward search for procedures if they had any episode with one of the procedures in the period April 2017 to March 2018. For each identified patient, the three-character primary diagnosis codes from all episodes in emergency admissions in the period April 2017 to March 2018 were captured. Primary diagnosis codes that occurred in at least 0.5% of patients were considered for inclusion in the algorithms.

For backward searching by cause of death, all deaths recorded by ONS with CLD as the underlying cause of death in the period 1 April 2017 to 20 September 2020 were examined. The underlying cause of death was defined as the disease or injury that initiated the train of events directly leading to death, and was coded using ICD-10 codes from the death certificate according to ONS guidelines.^32^ For each person with CLD as their underlying cause of death, HES records were examined to identify all emergency hospital admissions for that person in the period April 2015 to March 2019. The three-character primary diagnosis codes from all episodes in those emergency admissions were captured, and codes that occurred in at least 0.5% of admissions were considered for inclusion in the algorithms.

#### Code classification

Clinical experts (VB and WB) considered all codes identified through forward and backward searching and classified them into one of four groups: (A) presence alone indicates cirrhotic CLD; (B) presence alone indicates CLD but not cirrhosis; (C) presence alone does not indicate CLD, but is a common sign, symptom, complication, or cause of CLD; or (D) presence of code indicates that CLD is not the main reason for admission.

#### Algorithm specification

On the basis of these groups, three algorithms were defined for identifying emergency admissions for CLD, using the following principles (Table 1): (1) most specific algorithm: primary diagnosis must be CLD; (2) more sensitive (less specific) algorithm: primary diagnosis must be CLD, or a sign, symptom, complication, or cause of CLD, and CLD must be among the secondary diagnoses; and (3) most sensitive (least specific) algorithm: CLD can be a primary or secondary diagnosis, provided that there is an indication of advanced or severe disease, either through the presence of cirrhosis in any diagnosis field, or a sign, symptom, complication, or cause of CLD in any diagnosis field alongside the CLD diagnosis.

**Table 1:** Criteria of the most specific, more sensitive, and most sensitive algorithms.

Algorithm	Inclusion criteria	Exclusion criteria
Most specific algorithm	CLD code (group A or B) as the primary diagnosis field	Paracetamol poisoning (T39.1) in any diagnosis field
More sensitive algorithm	CLD code (group A or B) as the primary diagnosis field *OR* CLD-related code (group C) as the primary diagnosis field AND CLD code (group A or B) in any other diagnosis field	Paracetamol poisoning (T39.1) in any diagnosis field
Most sensitive algorithm	CLD code (group A or B) as the primary diagnosis field *OR* CLD-related code (group C) in any diagnosis field AND CLD code (group A or B) in any other diagnosis field *OR* Cirrhotic CLD code (group A) in any diagnosis field	Paracetamol poisoning (T39.1) in any diagnosis field *OR* Malignant neoplasm, not of the liver (C00–C21, C23–C97) as the primary diagnosis field

CLD, chronic liver disease.

The rationale for the more sensitive algorithm is that CLD can present in many ways and, therefore, signs and symptoms, such as ascites or haematemesis, may appear as the primary reason for the hospital admission, with CLD recorded in a secondary diagnosis field. The rationale for the most sensitive algorithm is that there can also be nonspecific presentations, which neither increase nor decrease the suspicion of CLD because they are associated with many diseases as well as CLD. These nonspecific presentations, such as sepsis or alcohol intoxication, might appear as the primary reason for the hospital admissions, with CLD and its signs and symptoms recorded in secondary diagnosis fields.

For all algorithms, patients were excluded if paracetamol poisoning was a primary or secondary diagnosis, because it is assumed that, even in a patient with CLD, the paracetamol poisoning is the reason for the acute admission rather than an acute worsening of CLD. For the most sensitive algorithm (in which the primary diagnosis was not necessarily CLD or CLD related), patients were excluded if their primary diagnosis was non-liver cancer, because this is assumed to decrease the likelihood that the primary reason for the admission was CLD and is the most common cause of ascites after CLD.^33^ A sensitivity analysis was carried out in which primary diagnoses of non-liver cancer (C00–C21 or C23–C97 as the primary diagnosis field) were not excluded from the most sensitive algorithm.

To identify patients for whom the emergency admission was their first hospital admission for CLD, patients were excluded if they had any previous admission (emergency or planned), as defined by that algorithm, during the preceding 5 years (or since April 2013 for patients without a full 5 years of preceding HES data). As a sensitivity analysis, we explored the effect of reducing the period for excluding previous CLD admissions to 2 years.

### Validation

Hospital admissions from April 2018 to March 2019 linked to ONS mortality data were used to validate the algorithms. Factors associated with CLD were compared between three groups: patients identified by the most specific algorithm, and the additional patients identified by the more and most sensitive algorithms.

Patient characteristics known to be predictive of, or associated with, CLD, treatments of CLD, outcomes of CLD, and the clinical specialty providing hospital care were compared between the three groups. Chi-squared tests were used to compare the distributions of these characteristics between the three groups.

Patient characteristics considered to be predictive of, or associated with, CLD were sex, age (grouped as 18–34, 35–49, 50–64, 65–79, and 80+ years of age), deprivation according to quintiles of the national IMD distribution, ethnicity, Royal College of Surgeons (RCS) Charlson comorbidity score, and individual comorbidities. Treatments considered were paracentesis and endoscopic bleeding treatments during the first emergency admission for CLD. Outcomes of CLD considered were all-cause mortality (for all deaths up to 20 September 2020), and underlying cause of death among those who died within a year of the first emergency admission.

Finally, the cohorts were compared with respect to the specialty of the consultant who treated the patient during their first emergency admission. Consultant specialty is recorded in two ways in HES data: the main specialty, under which the consultant was contracted, and the treatment specialty, under which the consultant worked. Main specialities were gastroenterology, internal or acute medicine (combined), and intensive care medicine or anaesthetics (combined). Treatment specialities were hepatology, gastroenterology, hepatology or gastroenterology (combined), internal medicine, and intensive care medicine or anaesthetics (combined). A patient’s care within the same admission is often transferred between consultants and, therefore, the proportion of patients under the care of each type of consultant often adds to more than 100%. As a proxy for admission to an ICU or HDU, the proportions of patients who underwent invasive ventilation during the first emergency admission were also compared.

We also assessed how the choice of algorithm affects the associations estimated between key patient characteristics and outcomes/treatment by an appropriate specialist. To do this, we fitted a series of logistic regression models for each of three binary outcomes: all-cause mortality within a year of the first emergency admission; death from CLD within a year of the first emergency admission; and treatment by a gastroenterologist or hepatologist during the first emergency admission. For the cohort identified using each of the three algorithms, we estimated the crude associations between the outcomes/treatment by an appropriate specialist and five patient characteristics: age, sex, ethnicity, number of comorbidities, and deprivation quintile.

## Results

**Fig. 1:**
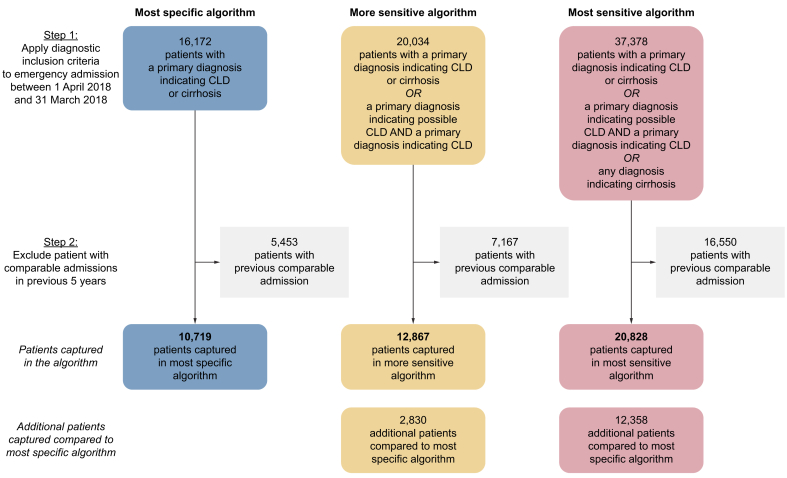
Flow chart describing the three algorithms used to identify patients with a first emergency admission for chronic liver disease. CLD, chronic liver disease.

Four-character ICD-10 codes identified through forward searching were classified into those that indicated cirrhosis, CLD, and signs, symptoms, causes, and complications of CLD (Table 2). The most specific algorithm identified 10,719 patients with a first emergency admission for CLD from April 2018 to March 2019 (Fig. 1). The more sensitive algorithm identified an additional 2,830 patients, and the most sensitive algorithm an additional 12,358 patients, each compared with the most specific algorithm.

**Table 2:** Classification of 4-character ICD-10 codes.

(A) Cirrhotic CLD codes		(B) Non-cirrhotic CLD codes		(C) CLD-related codes (signs, symptoms, complications, or causes of CLD)		(D) Rules out CLD	
Oesophageal varices with bleeding	I85.0	Chronic viral HBV with delta-agent	B18.0	HAV with hepatic coma	B15.0	Malignant neoplasm, not of liver	C00–C21, C23–C97
Oesophageal varices without bleeding	I85.9	Chronic viral HBV without delta-agent	B18.1	Liver cell carcinoma	C22.0	Poisoning by nonopioid analgesics, antipyretics and antirheumatics: 4-aminophenol derivatives	T39.1
Gastric varices	I86.4	Chronic viral HCV	B18.2	Intrahepatic bile duct carcinoma	C22.1		
Oesophageal varices in diseases classified elsewhere with bleeding	I98.2	Other chronic viral hepatitis	B18.8	Hepatoblastoma	C22.2		
Oesophageal varices in diseases classified elsewhere without bleeding	I98.3	Chronic viral hepatitis, unspecified	B18.9	Other specified carcinomas of liver	C22.7		
Alcoholic cirrhosis of liver	K70.3	Alcoholic fatty liver	K70.0	Liver, unspecified	C22.9		
Secondary biliary cirrhosis	K74.4	Alcoholic hepatitis	K70.1	Disorders of iron metabolism	E83.1		
Biliary cirrhosis, unspecified	K74.5	Alcoholic fibrosis and sclerosis of liver	K70.2	Disorders of plasma-protein metabolism, not classified elsewhere	E88.0		
Other and unspecified cirrhosis of liver	K74.6	Alcoholic hepatic failure	K70.4	Encephalopathy, unspecified	G93.4		
Portal hypertension	K76.6	Alcoholic liver disease, unspecified	K70.9	Acute peritonitis	K65.0		
Hepatorenal syndrome	K76.7	Toxic liver disease with fibrosis and cirrhosis of liver	K71.7	Peritonitis, unspecified	K65.9		
		Toxic liver disease with other disorders of liver	K71.8	Acute and subacute hepatic failure	K72.0		
		Chronic hepatic failure	K72.1	Hepatic failure, unspecified	K72.9		
		Chronic active hepatitis, not elsewhere classified	K73.2	Haematemesis	K92.0		
		Chronic hepatitis, unspecified	K73.9	Melaena	K92.1		
		Hepatic fibrosis	K74.0	Gastrointestinal haemorrhage, unspecified	K92.2		
		Hepatic sclerosis	K74.1	Unspecified jaundice	R17.X		
		Hepatic fibrosis with hepatic sclerosis	K74.2	Ascites	R18.X		
		Primary biliary cirrhosis	K74.3				
		Autoimmune hepatitis	K75.4				
		Other specified inflammatory liver diseases	K75.8				
		Inflammatory liver disease, unspecified	K75.9				

CLD, chronic liver disease; ICD-10, International Classification of Diseases, 10th revision.

### Backward searching

There were 25,358 deaths with CLD as the underlying cause in the period from 1 April 2017 to 20 September 2020, of whom 19,687 had had at least one episode of care associated with an emergency admission between April 2015 and March 2019, with a total of 178,773 hospital episodes. The diagnoses appearing in at least 0.5% of admissions are listed in Table S1. None of the diagnoses of CLD, signs, symptoms, causes, or complications of CLD had been missed in the forward searching step.

There were 23,931 patients who had paracentesis, 8,150 who had an endoscopic bleeding treatment, and 334 who had TIPS in the period April 2017 to March 2018. Of these, 21,428, 6,955, and 294, respectively had at least one episode of care associated with an emergency admission between April 2007 and March 2018. In total 155,700, 55,349, and 3,045 episodes were examined during emergency admissions preceding paracentesis, endoscopic bleeding treatment, and TIPS, respectively. The diagnoses appearing in at least 0.5% of admissions are listed in Tables S2–S4. Again, none of the diagnoses of CLD, signs, symptoms, causes, or complications of CLD had been missed in the forward searching step.

### Algorithm validation

Patient characteristics and CLD aetiologies are presented in Table 3. Additional patients identified by the more and most sensitive algorithms were more likely to be men (65.0%, *p* <0.001 and 62.1%, *p* = 0.008, respectively) than those identified by the most specific algorithm (60.0%). Patients identified by the more and most sensitive algorithms were generally older, with 44.1% of additional patients identified by the more sensitive algorithm, and 49.6% of those identified by the most sensitive algorithm, aged over 64 years, compared with 29.3% of those identified by the most specific algorithm. Patients identified by the more and most sensitive algorithms were also more likely to be from minority ethnic groups compared with those identified by the most specific algorithm: 7.2% identified by the most specific algorithm had an ethnic minority background, compared with 10.6% and 8.9% of those identify by the more and most sensitive algorithms, respectively. There were no differences with respect to deprivation quintiles between the groups. Patients identified by the more and most sensitive algorithms were less likely to have alcohol-related CLD and more likely to have no aetiology recorded during their first emergency admission for CLD.

**Table 3:** Characteristics of patients with a first emergency admission for CLD in April 2018-March 2019.

Characteristics	Most specific definition: CLD as primary diagnosis	Additional patients identified through more sensitive algorithm		Additional patients identified through most sensitive algorithm	
	N (%)	N (%)	*p* value∗	N (%)	*p* value∗
All patients	10,719	2,830		12,358	
Male	6,478 (60.4)	1,839 (65.0)	<0.001	7,675 (62.1)	0.009

**Age (years)**					
18–34	626 (5.8)	115 (4.1)	<0.001	426 (3.5)	<0.001
35–49	2,663 (24.8)	503 (17.8)		2,054 (16.6)	
50–64	4,293 (40.1)	963 (34.0)		3,857 (31.2)	
65–79	2,508 (23.4)	903 (31.9)		3,995 (32.3)	
80+	619 (5.9)	346 (12.2)		2,016 (16.3)	
**% in neighbourhood deprivation quintiles**					
Wealthiest	1,397 (13.3)	362 (13.2)	0.376	1,501 (12.6)	0.518
Wealthier	1,647 (15.7)	441 (16.1)		1,881 (15.8)	
Middle	2,003 (19.1)	480 (17.5)		2,315 (19.4)	
Poorer	2,399 (22.9)	635 (23.1)		2,703 (22.7)	
Poorest	3,049 (29.1)	829 (30.2)		3,539 (29.6)	
**Ethnic background**					
White	8,937 (83.4)	2,296 (81.1)	<0.001	10,270 (83.1)	<0.001
Mixed/multiple	48 (0.5)	16 (0.6)		66 (0.5)	
Asian	401 (3.7)	152 (5.4)		584 (4.7)	
Black	127 (1.2)	66 (2.3)		233 (1.9)	
Other	188 (1.8)	66 (2.3)		221 (1.8)	
Not known/missing	1,018 (9.5)	234 (8.3)		984 (8.0)	
**Aetiology of CLD**†**(not mutually exclusive or collectively exhaustive)**					
Alcohol	7,515 (70.1)	1,154 (40.8)	<0.001	4,001 (32.4)	<0.001
Viral	469 (4.4)	349 (12.3)	<0.001	1,061 (8.6)	<0.001
Metabolic	1,375 (12.8)	358 (12.7)	0.801	1,198 (9.7)	<0.001
Autoimmune	373 (3.5)	99 (3.5)	0.962	244 (2.0)	<0.001
Other specified	490 (4.6)	140 (4.9)	0.399	683 (5.5)	0.001
None identified	1,881 (17.5)	933 (33.0)	<0.001	5,990 (48.5)	<0.001

CLD, chronic liver disease.

∗ From *χ*^2^ test for difference in proportions compared to most specific algorithm.

† see Table S5 for details and ICD-10 codes of aetiologies.

### Sensitivity analysis

A sensitivity analysis was carried out in which primary diagnoses of non-liver cancer were included in the most sensitive algorithm. This reduced the number of patients identified per year using the most sensitive algorithm by 0.6% (129/20,828 patients).

Table 4 compares the prevalence of comorbidities across the cohorts. Additional patients identified by the more sensitive algorithms had more comorbidities, with 66.4% (more sensitive algorithm) and 60.9% (most sensitive algorithm) having at least one comorbidity recorded in the first emergency admission or preceding year, compared with 51.2% in the cohort of patients identified with the most specific algorithm. The additional patients identified by the more and most sensitive algorithms had a higher prevalence of every individual comorbidity except dementia.

**Table 4:** Prevalence of comorbidities in patients with a first emergency admission for CLD from April 2018 to March 2019.

Characteristics	Most specific definition: CLD as primary diagnosis	Additional patients identified through more sensitive algorithm		Additional patients identified through most sensitive algorithm	
	N (%)	N (%)	*p* value∗	N (%)	*p* value∗
All patients	10,719	2,830		12,358	

**Charlson score for number of comorbidities**					
0	5,232 (48.8)	959 (33.9)	<0.001	3,597 (29.1)	<0.001
1	3,058 (28.5)	823 (29.1)		3,545 (28.7)	
2	1,375 (12.8)	524 (18.5)		2,417 (19.6)	
3+	1,054 (9.8)	524 (18.5)		2,799 (22.7)	
**Individual comorbidities**					
Myocardial infarction	516 (4.8)	229 (8.1)	<0.001	1,152 (9.3)	<0.001
Congestive cardiac failure	1,081 (10.1)	429 (15.2)	<0.001	2,595 (21.0)	<0.001
Peripheral vascular disease	459 (4.3)	234 (8.3)	<0.001	1,139 (9.2)	<0.001
Cerebrovascular disease	510 (4.8)	196 (7.0)	<0.001	1,107 (9.0)	<0.001
Dementia	758 (7.1)	194 (6.9)	0.689	1,030 (8.3)	<0.001
Chronic pulmonary disease	2,335 (21.8)	722 (25.5)	<0.001	3,815 (30.9)	<0.001
Rheumatological disease	270 (2.5)	3.4 (3.4)	0.014	531 (4.3)	<0.001
Diabetes mellitus	2,033 (19.0)	826 (29.3)	<0.001	3,584 (29.0)	<0.001
Hemiplegia or paraplegia	95 (0.9)	42 (1.5)	0.005	228 (1.8)	<0.001
Renal disease	740 (6.9)	388 (13.7)	<0.001	2,001 (16.2)	<0.001
Any malignancy (excluding liver and intrahepatic bile ducts)	508 (4.7)	260 (9.2)	<0.001	1,149 (9.3)	<0.001
Metastatic solid tumour	257 (2.4)	217 (7.8)	<0.001	557 (4.5)	<0.001

CLD, chronic liver disease.

∗ From χ^2^ test for difference in proportions compared with most specific algorithm.

Procedures for AdvCLD are shown in Table 5. Of patients identified by the most specific algorithm, 34.3% had a paracentesis in the first emergency admission, and 10.2% an endoscopic treatment for bleeding. These were significantly lower among the additional patients identified by the more sensitive algorithm, at 22.0% and 3.3%, respectively, and lower still in the additional patients identified through most sensitive algorithm, at 9.1% and 1.5%, respectively.

**Table 5:** Procedures for AdvCLD during the first emergency admission for CLD in April 2018-March 2019.

Procedure	Most specific algorithm: CLD as primary diagnosis	Additional patients identified through more sensitive algorithm		Additional patients identified through most sensitive algorithm	
	N (%)	N (%)	*p* value∗	N (%)	*p* value∗
All patients	10,719	2,830		12,358	
Paracentesis	3,685 (34.4)	622 (22.0)	<0.001	1,120 (9.1)	<0.001
Endoscopic bleeding treatments	1,092 (10.2)	93 (3.3)	<0.001	179 (1.5)	<0.001

AdvCLD, advanced chronic liver disease; CLD, chronic liver disease.

∗ From *χ*^2^ test for difference in proportions compared with most specific algorithm.

**Fig. 2:**
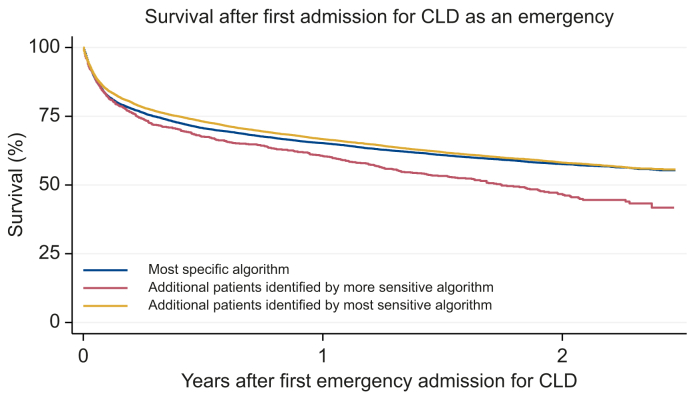
Survival after a first emergency admission for chronic liver disease between April 2018 and March 2019.

Additional patients identified by the more sensitive algorithm had poorer survival compared with the patients identified through the most specific algorithm (Fig. 2). Of those who died within 1 year of the first emergency admission, a lower proportion of additional patients identified by the more sensitive algorithm had CLD (31.0% *vs*. 59.9%) or other non-cancer liver disease (4.0% *vs.* 6.3%) as the underlying cause of death (Table 6). Notably, 26.1% of the additional patients identified by the more sensitive algorithm who died within 1 year had liver cancer as the underlying cause of death, compared with 5.8% of the cohort defined according to the most specific algorithm.

**Table 6:** Causes of death among patients who died within 1 year of a first emergency admission for CLD from April 2018 to March 2019.

Characteristics	Most specific algorithm: CLD as primary diagnosis	Additional patients identified through more sensitive algorithm		Additional patients identified through most sensitive algorithm	
	N (%)	N (%)	*p* value∗	N (%)	*p* value∗
All patients	10,719	2,830		12,358	
Deaths within 1 year	3,757 (35.1)	1,142 (40.4)	<0.001	4,149 (33.6)	0.018

**Of those who died: underlying cause of death**					
Cirrhosis and non-cirrhotic CLD	2,251 (59.9)	354 (31.0)	<0.001	831 (20.0)	<0.001
Other liver diseases (excluding liver cancer)	235 (6.3)	46 (4.0)		125 (3.0)	
Other diseases of digestive system	109 (2.9)	51 (4.5)		250 (6.0)	
Liver cancer	219 (5.8)	298 (26.1)		384 (9.3)	
Other cancer	216 (5.8)	135 (11.8)		617 (14.9)	
Diseases of circulatory system	260 (6.9)	86 (7.5)		734 (17.7)	
Diseases of respiratory system	133 (3.5)	46 (4.0)		445 (10.7)	
All other causes	345 (9.2)	129 (11.3)		769 (18.5)	

CLD, chronic liver disease.

∗ From *χ*^2^ test for difference in proportions compared to most specific algorithm.

Additional patients identified by the most sensitive algorithm had similar mortality to those identified by the most specific algorithm. Of those who died during the first year after admission, a lower proportion (compared with both the most specific and more sensitive algorithms) had CLD (20.0%) or other non-cancer liver disease (3.0%) as the underlying cause of death, and 9.3% had liver cancer as the underlying cause.

Table 7 shows that 93.3% of patients identified by the most specific algorithm were under the care of a consultant working under the specialty of hepatology, gastroenterology, internal medicine, intensive care, or anaesthetics. The additional patients identified by the more sensitive and most sensitive algorithms were less likely to be under the care of a specialist appropriate for the treatment of CLD (a hepatologist or gastroenterologist). There was no difference in the proportion of patients under the care of an anaesthetic or intensive care specialist, or in the proportion of those who underwent invasive ventilation.

**Table 7:** Proportion of patients under care of clinical specialists during the first emergency admission for CLD from April 2018 to March 2019.

	Most specific algorithm: CLD as primary diagnosis	Additional patients identified through more sensitive algorithm		Additional patients identified through most sensitive algorithm	
	N (%)	N (%)	*p* value∗	N (%)	*p* value∗
All patients	10,719	2,830		12,358	

**Main specialty (specialty under which consultant contracted)**†					
Gastroenterology	5,789 (54.0)	1,291 (45.6)	<0.001	9,134 (26.1)	<0.001
Internal or acute medicine	7,481 (69.8)	7,073 (63.0)	<0.001	7,073 (57.2)	<0.001
Intensive care or anaesthetics	292 (2.7)	71 (2.5)	0.582	347 (2.8)	0.631
None of the above	944 (8.8)	488 (17.2)	<0.001	3,720 (30.1)	<0.001

**Treatment specialty (specialty under which consultant worked)**†					
Hepatology	1,012 (9.4)	204 (7.2)	<0.001	432 (3.5)	<0.001
Gastroenterology	5,551 (51.8)	1,229 (43.4)	<0.001	2,918 (23.6)	<0.001
Hepatology or gastroenterology	6,272 (58.5)	1,392 (49.2)	<0.001	3,250 (26.3)	<0.001
Internal medicine	8,179 (76.3)	7,871 (69.4)	<0.001	7,871 (63.7)	<0.001
Intensive care or anaesthetics	175 (1.6)	51 (1.8)	0.463	211 (1.7)	0.574
None of the above	714 (6.7)	402 (14.2)	<0.001	3,349 (27.1)	<0.001

**Critical care**					
Invasive ventilation‡	390 (3.6)	97 (3.4)	0.594	475 (3.8)	0.418

∗ From *χ*^2^ test for difference in proportions compared with most specific algorithm.

† Specialities sum to more than 100% because patients can be under the care of more than one consultant during their admission. These patients have more than episode of care during their admission.

‡ Invasive ventilation is a proxy for intensive care admission.

**Fig. 3:**
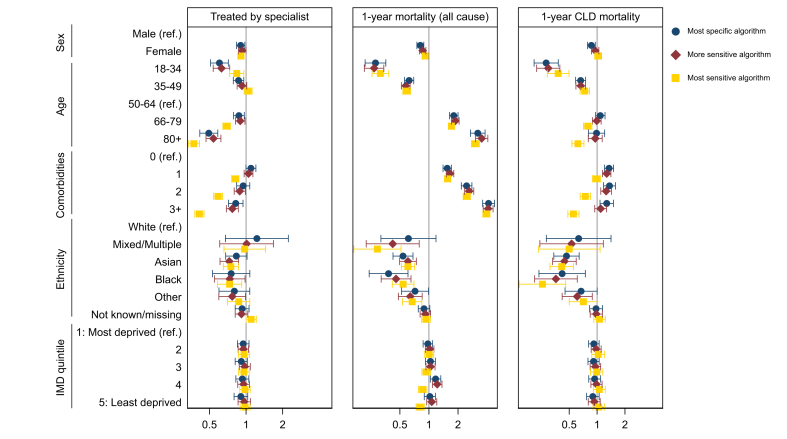
Associations between outcomes/treatment by an appropriate specialist (hepatologist or gastroenterologist) and patient characteristics in the cohorts identified by each of the three algorithms. The comparison is between all patients captured in each algorithm (rather than comparing additional patients captured in the more and most sensitive algorithms). CLD, chronic liver disease; IMD, Index of Multiple Deprivation.

Results of logistic regression modelling comparing the use of the most specific, the more sensitive, and the most sensitive algorithms to examine univariate associations between patient characteristics and outcomes/treatment by an appropriate specialist are shown in Fig. 3. Here, the comparison is between all patients captured in each algorithm (rather than comparing patients captured in the most specific algorithm to the additional patients captured in the more and most sensitive algorithms). The results demonstrate that the associations do not vary according to the algorithm used, with the exception of number of comorbidities, which has a more marked association with both 1-year CLD mortality and treatment by an appropriate specialist when the most sensitive algorithm is used to define the cohort.

Finally, in a sensitivity analysis, we explored the effect of reducing the period for excluding previous CLD admissions to 2 years. This increased the number of patients captured by the most sensitive algorithm by 1,389 (an increase of 12.9%).

## Discussion

Using a comprehensive process of ‘forward searching’ for codes drawn from existing literature and expert clinical opinion, followed by a data-driven ‘backward searching’ step to identify additional unpredictable idiosyncrasies of coding practices, we have developed three algorithms to identify patients with a first emergency admission for CLD.

Our validation demonstrates the extent to which the more sensitive algorithms become more inclusive at the expense of a reduced specificity. The choice of algorithm will need to be made with the required balance of sensitivity and specificity in mind, which is guided by the relative importance of erroneously including or erroneously excluding patients. The most specific approach, which aims to keep the number of erroneously included patients low, identified ∼10,000 patients admitted to English NHS hospitals in 1 year. Among patients identified using this approach, 93% were under the care of a specialist appropriate for the treatment of CLD (a hepatologist or gastroenterologist), and most deaths were from CLD (60% of the deaths within 1 year were from CLD, rising to 72% when including other liver diseases and liver cancer).

The cohort size expanded by 26% with the use of the more sensitive algorithm and by 115% with the use of the most sensitive algorithm. Within these larger cohorts, a higher proportion of deaths were from non-liver causes, a lower proportion of patients were under the care of gastroenterologists or general internal medicine specialists, and patients tended to have more comorbidities. While the most specific algorithm undoubtedly excludes some first emergency hospital admissions for CLD (*i.e.* erroneous exclusions), the more and most sensitive algorithms inevitably capture some patients admitted to hospital with CLD (*i.e.* erroneous inclusions). Reassuringly, although the estimated number of identified patients was very sensitive to the choice of algorithm, the associations between key outcomes and patient characteristics changed very little when different algorithms were used to identify the patients in the cohorts.

### Strengths and limitations

A particular strength of the development of these algorithms is the use of national administrative data for all admissions to all NHS hospitals in England. This means that, at a national level, the results are robust to variation and idiosyncrasies in coding practices between hospitals. We have not, by design, validated algorithms against patients’ full medical records to verify whether a CLD diagnosis was made during the admission, since this would only be possible in very small numbers at a limited selection of hospitals. We assumed that patients with no hospital admission (emergency or otherwise) for CLD in the preceding 5 years were presenting for the first time with CLD. There will be a small number of patients who had a diagnosis of CLD before this who our algorithms will incorrectly include as first emergency admissions. However, among patients with CLD severe enough to require emergency admission, only a small proportion would be expected to have no hospital admissions (overnight or day-case) in 5 years. Finally, in the hospital administrative dataset, patients are assigned to one senior clinical specialist for each episode of care. This means that we were only able to measure the proportion of patients under the care of an appropriate specialist. The proportion of patients seen by an appropriate specialist is likely to be higher.

### Comparison with previous literature

This is, to our knowledge, the first study to develop and validate algorithms with varying levels of specificity and sensitivity to identify patients with CLD first diagnosed during an emergency admission. An algorithm has been developed to identify all (not only first) emergency admissions for alcohol-related liver disease.^34^ Including secondary diagnostic codes in this algorithm has been demonstrated to capture more than double the number of patients, compared with the standard approach of using primary diagnoses only.^34^ A more recent study implemented this algorithm to identify first emergency admissions for alcohol-related liver disease and estimated that there were 2,000 first admissions per year between April 2013 and March 2018.^35^ Our algorithms, which were developed using a comprehensive process to capture all aetiologies of CLD, demonstrate that the burden of first emergency admissions for all types of CLD is much higher.

### Recommendations

The more and most sensitive algorithms are recommended in studies when it is important to minimise the number of patients with CLD erroneously missed from the cohort, and when it is acceptable that some of the patients included have CLD as a comorbidity rather than as the main clinical presentation. An example of such a study would be measuring the burden of disease, when researchers will want to avoid underestimating the number of emergency admissions for CLD.

The risk of using the most specific algorithm is that, through requiring that CLD is recorded as the primary reason for admission, it will underestimate the true number of first emergency admissions for CLD, and will tend to miss patients with CLD with more comorbidities. However, we would recommend using the most specific algorithm when the interest is strictly in patients whose primary reason for the emergency admission is CLD.

For studies of the associations between risk factors and outcomes of CLD, or for developing risk prediction models for outcomes of CLD, it is reasonable to use the more sensitive algorithms, because the associations between patient characteristics and outcomes were consistent across algorithms, and this will lead to a statistically more powerful analysis.

Whatever the purpose of the study and the initial algorithm chosen to identify the patients, it is recommended that sensitivity analyses are carried out to assess whether the study results are substantially different when other algorithms are used.

## Abbreviations

AdvCLD, advanced CLD; APC, admitted patient care; CLD, chronic liver disease; HDU, high-dependency unit; HES, Hospital Episode Statistics; ICD-10, International Classification of Diseases, 10th revision; ICU, intensive care unit; IMD, Index of Multiple Deprivation; NHS, National Health Service; ONS, Office for National Statistics; OPCS-4, Office of Population Censuses and Surveys Classification of Surgical Operations and Procedures, 4th revision; RCS, Royal College of Surgeons; TIPS, transjugular intrahepatic portosystemic shunt.

## Financial support

This research was funded by a grant from the National Institute for Health and Care Research (NIHR 132969).

## Authors’ contributions

Conceptualisation (lead): KW. Conceptualisation (equal); JK, WB, JVDM. Conceptualisation (supporting): VB, JD. Methodology (lead): JVDM. Methodology (equal): JK, KW. Methodology (supporting): VB, JD. Formal analysis (lead): JK. Writing – original draft (lead): JK. Writing – original draft (equal): JVDM, KW, WB. Writing – review and editing (lead): KW. Writing – review and editing (equal): JK, VB, JD, JVDM, WB. Funding acquisition (lead): WB. Funding acquisition (equal): JVDM, KW.

## Data availability statement

This study uses Hospital Episode Statistics from NHS England, which can be requested via the Data Access Request Service (https://digital.nhs.uk/services/data-access-request-service-dars).

## Conflicts of interest

The authors of this study declare that they do not have any conflict of interest.

Please refer to the accompanying ICMJE disclosure forms for further details.

## References

[1] Williams R., Aspinall R., Bellis M. (2014). Addressing liver disease in the UK: a blueprint for attaining excellence in health care and reducing premature mortality from lifestyle issues of excess consumption of alcohol, obesity, and viral hepatitis. Lancet.

[2] Public Health England (2017).

[3] Office for National Statistics (2020).

[4] Asrani S.K., Kamath P.S. (2013). Natural history of cirrhosis. Curr Gastroenterol Rep.

[5] Shah N.D., Ventura-Cots M., Abraldes J.G. (2019). Alcohol-related liver disease is rarely detected at early stages compared with liver diseases of other etiologies worldwide. Clin Gastroenterol Hepatol.

[6] Roberts S.E., John A., Brown J. (2019). Early and late mortality following unscheduled admissions for severe liver disease across England and Wales. Aliment Pharmacol Ther.

[7] The National Confidential Enquiry into Patient Outcome and Death (NCEPOD) (2022).

[8] Berry P.A., Peck M., Standley T. (2016). Do critically ill liver patients experience negative bias? A web-based survey examining doctors opinions to critical care escalation. Frontline Gastroenterol.

[9] Williams R., Alessi C., Alexander G. (2021). New dimensions for hospital services and early detection of disease: a review from the Lancet Commission into liver disease in the UK. Lancet.

[10] McPhail M.J.W., Parrott F., Wendon J.A. (2018). Incidence and outcomes for patients with cirrhosis admitted to the United Kingdom critical care units. Crit Care Med.

[11] Gavrielov-Yusim N., Friger M. (2014). Use of administrative medical databases in population-based research. J Epidemiol Community Health.

[12] Bright R.A., Avorn J., Everitt D.E. (1989). Medicaid data as a resource for epidemiologic studies: strengths and limitations. J Clin Epidemiol.

[13] Lamoreaux J. (1996). The organizational structure for medical information management in the Department of Veterans Affairs: an overview of major health care databases. Med Care.

[14] Downey W., Stang M., Beck P., Strom B.L. (2006). Pharmacoepidemiology.

[15] Herbert A., Wijlaars L., Zylbersztejn A. (2017). Data resource profile: hospital episode statistics admitted patient care (HES APC). Int J Epidemiol.

[16] Jepsen P. (2014). Comorbidity in cirrhosis. World J Gastroenterol.

[17] NHS England Terminology and Classifications Delivery Service (2022).

[18] NHS England (2007).

[19] Armitage J.N., van der Meulen J.H. (2010). Identifying co-morbidity in surgical patients using administrative data with the Royal College of Surgeons Charlson Score. Br J Surg.

[20] Ministry of Housing Communities & Local Government (2015).

[21] Shearer J.E., Gonzalez J.J., Min T. (2022). Systematic review: development of a consensus code set to identify cirrhosis in electronic health records. Aliment Pharmacol Ther.

[22] Thygesen S.K., Christiansen C.F., Christensen S. (2011). The predictive value of ICD-10 diagnostic coding used to assess Charlson comorbidity index conditions in the population-based Danish National Registry of Patients. BMC Med Res Methodol.

[23] Fialla A.D., de Muckadell O.B.S., Touborg Lassen A. (2012). Incidence, etiology and mortality of cirrhosis: a population-based cohort study. Scand J Gastroenterol.

[24] Ratib S., Fleming K.M., Crooks C.J. (2014). 1 and 5 year survival estimates for people with cirrhosis of the liver in England, 1998–2009: a large population study. J Hepatol.

[25] Lu M., Chacra W., Rabin D. (2017). Validity of an automated algorithm using diagnosis and procedure codes to identify decompensated cirrhosis using electronic health records. Clin Epidemiol.

[26] Lapointe-Shaw L., Georgie F., Carlone D. (2018). Identifying cirrhosis, decompensated cirrhosis and hepatocellular carcinoma in health administrative data: a validation study. PLoS One.

[27] Driver R.J., Balachandrakumar V., Burton A. (2019). Validation of an algorithm using inpatient electronic health records to determine the presence and severity of cirrhosis in patients with hepatocellular carcinoma in England: an observational study. BMJ Open.

[28] Hayward K.L., Johnson A.L., Mckillen B.J. (2020). ICD-10-AM codes for cirrhosis and related complications: key performance considerations for population and healthcare studies. BMJ Open Gastroenterol.

[29] Dahiya M., Eboreime E., Hyde A. (2022). International classification of diseases codes are useful in identifying cirrhosis in administrative databases. Dig Dis Sci.

[30] Ratib S., West J., Fleming K.M. (2017). Liver cirrhosis in England—an observational study: are we measuring its burden occurrence correctly?. BMJ Open.

[31] Elixhauser A., Steiner C., Harris D.R. (1998). Comorbidity measures for use with administrative data. Med Care.

[32] Office for National Statistics (2022).

[33] Aithal G.P., Palaniyappan N., China L. (2021). Guidelines on the management of ascites in cirrhosis. Gut.

[34] Dhanda A., Bodger K., Hood S. (2023). The Liverpool alcohol-related liver disease algorithm identifies twice as many emergency admissions compared to standard methods when applied to Hospital Episode Statistics for England. Aliment Pharmacol Ther.

[35] Bodger K., Mair T., Schofield P. (2023). Outcomes of first emergency admissions for alcohol-related liver disease in England over a 10-year period: retrospective observational cohort study using linked electronic databases. BMJ Open.

