# Supplementary material for: Identifying emergency presentations of chronic liver disease using routinely collected administrative hospital data
Source: JHEP Rep. 2024 Dec 31;7(5):101322. doi: 10.1016/j.jhepr.2024.101322 (PMC12008566; doi:10.1016/j.jhepr.2024.101322)
Supplement: Multimedia component 1 [file mmc1.pdf]

Identifying emergency presentations of chronic liver disease using routinely collected administrative hospital data

Jessica King, Vikram Bains, James Doidge, Jan Van Der Meulen, Kate Walker, William Bernal

Table of contents

Table S1……………………………………………………………………………………2
Table S2……………………………………………………………………………………2
Table S3……………………………………………………………………………………3
Table S4……………………………………………………………………………………4
Table S5……………………………………………………………………………………4

Table S1: Common primary diagnoses in emergency admissions before death from CLD cause, by 3-character ICD-10 code

ICD-10 code	Primary diagnosis	Number of episodes	%
K70	Alcoholic liver disease	40,002	22.4
F10	Mental and behavioural disorders due to use of alcohol	12,536	7.0
K92	Other diseases of digestive system	7,364	4.1
J18	Pneumonia, organism unspecified	5,035	2.8
A41	Other sepsis	4,686	2.6
K72	Hepatic failure, not elsewhere classified	4,649	2.6
R18	Ascites	4,373	2.5
K74	Fibrosis and cirrhosis of liver	4,228	2.4
E87	Other disorders of fluid, electrolyte and acid-base balance	2,791	1.6
I98	Other disorders of circulatory system in diseases classified elsewhere	2,764	1.6
I85	Oesophageal varices	2,761	1.5
N39	Other disorders of urinary system	2,578	1.4
N17	Acute renal failure	2,568	1.4
R10	Abdominal and pelvic pain	2,275	1.3
L03	Cellulitis	2,235	1.3
K76	Other diseases of liver	1,827	1.0
I50	Heart failure	1,652	0.9
K65	Peritonitis	1,637	0.9
A09	Other gastroenteritis and colitis of infectious and unspecified origin	1,626	0.9
D64	Other anaemias	1,589	0.9
R29	Other symptoms and signs involving the nervous and musculoskeletal systems	1,552	0.9
J44	Other chronic obstructive pulmonary disease	1,541	0.9
K29	Gastritis and duodenitis	1,501	0.8
R07	Pain in throat and chest	1,484	0.8
K75	Other inflammatory liver diseases	1,461	0.8
K85	Acute pancreatitis	1,397	0.8
R56	Convulsions, not elsewhere classified	1,356	0.8
R55	Syncope and collapse	1,345	0.8
D50	Iron deficiency anaemia	1,274	0.7
J22	Unspecified acute lower respiratory infection	1,160	0.7
R41	Other symptoms and signs involving cognitive functions and awareness	1,091	0.6
R69	Unknown and unspecified causes of morbidity	1,078	0.6
S72	Fracture of femur	1,027	0.6
K22	Other diseases of oesophagus	995	0.6
S01	Open wound of head	950	0.5
J90	Pleural effusion, not elsewhere classified	947	0.5
T39	Poisoning by nonopioid analgesics, antipyretics and antirheumatics	870	0.5
E11	Type 2 diabetes mellitus	843	0.5
S00	Superficial injury of head	835	0.5
M79	Other soft tissue disorders, not elsewhere classified	828	0.5

Total number of episodes was 178773, only diagnosis codes which represent at least 0.5% of all episodes shown

Table S2: Common primary diagnoses in emergency admissions before paracentesis, by 3-character ICD-10 code

ICD-10 code	Primary diagnosis	Number of episodes	%
K70	Alcoholic liver disease	30884	19.8
R18	Ascites	8855	5.7
C78	Secondary malignant neoplasm of respiratory and digestive organs	6217	4.0
A41	Other sepsis	5602	3.6
K92	Other diseases of digestive system	4308	2.8
F10	Mental and behavioural disorders due to use of alcohol	3784	2.4
K74	Fibrosis and cirrhosis of liver	3665	2.4
J18	Pneumonia, organism unspecified	3192	2.1
I50	Heart failure	3010	1.9
K72	Hepatic failure, not elsewhere classified	2962	1.9
R10	Abdominal and pelvic pain	2459	1.6

C56	Malignant neoplasm of ovary	2453	1.6
N17	Acute renal failure	2235	1.4
K65	Peritonitis	2036	1.3
I85	Oesophageal varices	1931	1.2
E87	Other disorders of fluid, electrolyte and acid-base balance	1884	1.2
I98	Other disorders of circulatory system in diseases classified elsewhere	1861	1.2
C25	Malignant neoplasm of pancreas	1738	1.1
C22	Malignant neoplasm of liver and intrahepatic bile ducts	1670	1.1
J90	Pleural effusion, not elsewhere classified	1596	1.0
K76	Other diseases of liver	1540	1.0
N39	Other disorders of urinary system	1539	1.0
K75	Other inflammatory liver diseases	1417	0.9
A09	Other gastroenteritis and colitis of infectious and unspecified origin	1365	0.9
K83	Other diseases of biliary tract	1318	0.9
K85	Acute pancreatitis	1269	0.8
D64	Other anaemias	1122	0.7
L03	Cellulitis	1089	0.7
K56	Paralytic ileus and intestinal obstruction without hernia	1039	0.7
C80	Malignant neoplasm, without specification of site	987	0.6
R07	Pain in throat and chest	974	0.6
K59	Other functional intestinal disorders	970	0.6
C18	Malignant neoplasm of colon	956	0.6
R11	Malignant neoplasm of nasopharynx	918	0.6
T81	Complications of procedures, not elsewhere classified	840	0.5
J22	Unspecified acute lower respiratory infection	834	0.5
K29	Gastritis and duodenitis	756	0.5
D50	Iron deficiency anaemia	750	0.5
J44	Other chronic obstructive pulmonary disease	745	0.5
I26	Pulmonary embolism	741	0.5
K86	Other diseases of pancreas	716	0.5

Total number of episodes was 15570, only diagnosis codes which represent at least 0.5% of all episodes shown

Table S3: Common primary diagnoses in emergency admissions before endoscopic bleeding treatment, by 3-character ICD-10 code

ICD-10 code	Primary diagnosis	Number of episodes	%
K70	Alcoholic liver disease	7049	12.7
K92	Other diseases of digestive system	5430	9.8
I85	Oesophageal varices	3976	7.2
I98	Other disorders of circulatory system in diseases classified elsewhere	3034	5.5
K25	Gastric ulcer	2050	3.7
F10	Mental and behavioural disorders due to use of alcohol	1547	2.8
K22	Other diseases of oesophagus	1455	2.6
J18	Pneumonia, organism unspecified	1283	2.3
A41	Other sepsis	1199	2.2
K26	Duodenal ulcer	1130	2.0
R18	Ascites	965	1.7
K74	Fibrosis and cirrhosis of liver	930	1.7
R10	Abdominal and pelvic pain	785	1.4
D64	Other anaemias	734	1.3
N39	Other disorders of urinary system	667	1.2
K72	Hepatic failure, not elsewhere classified	659	1.2
D50	Iron deficiency anaemia	638	1.2
I50	Heart failure	582	1.1
N17	Acute renal failure	543	1.0
K31	Other diseases of stomach and duodenum	499	0.9
I86	Varicose veins of other sites	482	0.9
R07	Pain in throat and chest	474	0.9
J44	Other chronic obstructive pulmonary disease	466	0.8
A09	Other gastroenteritis and colitis of infectious and unspecified origin	438	0.8
E87	Other disorders of fluid, electrolyte and acid-base balance	437	0.8

K29	Gastritis and duodenitis	408	0.7
L03	Cellulitis	374	0.7
K76	Other diseases of liver	372	0.7
I21	Acute myocardial infarction	359	0.7
J22	Unspecified acute lower respiratory infection	335	0.6
R29	Other symptoms and signs involving the nervous and musculoskeletal systems	318	0.6
K65	Peritonitis	299	0.5
S72	Fracture of femur	271	0.5
R55	Syncope and collapse	262	0.5

Total number of episodes was 55349, only diagnosis codes which represent at least 0.5% of all episodes shown

Table S4: Common primary diagnoses in emergency admissions before TIPS, by 3-character ICD-10 code

ICD-10 code	Primary diagnosis	Number of episodes	%
K70	Alcoholic liver disease	814	26.7
I98	Other disorders of circulatory system in diseases classified elsewhere	190	6.2
K92	Other diseases of digestive system	169	5.6
R18	Ascites	164	5.4
I85	Oesophageal varices	158	5.2
K72	Hepatic failure, not elsewhere classified	140	4.6
I86	Varicose veins of other sites	89	2.9
K74	Fibrosis and cirrhosis of liver	78	2.6
A41	Other sepsis	73	2.4
F10	Mental and behavioural disorders due to use of alcohol	57	1.9
I82	Other venous embolism and thrombosis	49	1.6
K22	Other diseases of oesophagus	40	1.3
T81	Complications of procedures, not elsewhere classified	37	1.2
E87	Other disorders of fluid, electrolyte and acid-base balance	35	1.2
R10	Abdominal and pelvic pain	33	1.1
K76	Other diseases of liver	32	1.1
N17	Acute renal failure	32	1.1
K65	Peritonitis	30	1.0
K75	Other inflammatory liver diseases	30	1.0
K91	Postprocedural disorders of digestive system, not elsewhere classified	28	0.9
K42	Umbilical hernia	25	0.8
J90	Pleural effusion, not elsewhere classified	24	0.8
I81	Portal vein thrombosis	21	0.7
K83	Other diseases of biliary tract	21	0.7
K59	Other functional intestinal disorders	20	0.7
D64	Other anaemias	18	0.6
D50	Iron deficiency anaemia	16	0.5
G93	Other disorders of brain	15	0.5
J18	Pneumonia, organism unspecified	14	0.5
J94	Other pleural conditions	14	0.5
R41	Other symptoms and signs involving cognitive functions and awareness	14	0.5
S72	Fracture of femur	14	0.5

Total number of episodes was 3045, only diagnosis codes which represent at least 0.5% of all episodes shown

Table S5: CLD aetiology groups and ICD-10 codes

Aetiology group	Diagnoses	ICD-10 codes*
Alcohol	Alcoholic liver disease (including alcoholic fatty liver, alcoholic hepatitis, alcoholic fibrosis and sclerosis of liver, alcoholic cirrhosis of liver, alcoholic hepatic failure and alcoholic liver disease, unspecified	K70.0, K70.1, K70.2, K70.3, K70.4, K70.9
Viral	Chronic viral hepatitis (including chronic viral hepatitis B with delta-agent, chronic viral hepatitis B without delta-agent, chronic viral hepatitis C, other chronic viral hepatitis, and chronic viral hepatitis, unspecified)	B18.0, B18.1, B18.2, B18.8, B18.9
Metabolic	Metabolic dysfunction-associated steatotic liver disease (MASLD), Metabolic dysfunction-associated steatohepatitis (MASH)	K75.8, K76.0

Autoimmune	Primary biliary cirrhosis<br>Autoimmune hepatitis	K74.3, K75.4
Other specified	Disorders of tyrosine metabolism (Alkaptonuria, Hypertyrosinaemia, Ochronosis, Tyrosinaemia, Tyrosinosis), Glycogen storage disease, Other specified disorders of carbohydrate metabolism (Essential pentosuria, Oxalosis, Oxaluria, Renal glycosuria), Other sphingolipidosis, Crigler-Najjar syndrome, Disorders of copper metabolism, Disorders of iron metabolism (Haemochromatosis), Amyloidosis, Disorders of plasma-protein metabolism, not elsewhere classified (Alpha-1-antitrypsin deficiency, Bisalbuminaemia), Toxic liver disease, Budd-Chiari syndrome, Other specified diseases of biliary tract	E70.2, E74.0, E74.8, E75.2, E80.5, E83.0, E83.1, E85.X, E88.0, K71.X, I82.0, K83.8

*For ICD-10 codes with 3 characters followed by ".X", all sub-codes of that 3-character code are included
